# Self-reports from behind the scenes: Questionable research practices and rates of replication in ego depletion research

**DOI:** 10.1371/journal.pone.0199554

**Published:** 2018-06-25

**Authors:** Wanja Wolff, Lorena Baumann, Chris Englert

**Affiliations:** 1 Department of Sport Psychology, University of Konstanz, Konstanz, Germany; 2 Department of Educational Psychology, University of Bern, Bern, Switzerland; Universidad de Granada, SPAIN

## Abstract

The strength model of self-control is one of the most influential and well-established models of self-regulation in social psychology. However, recent attempts to replicate the ego depletion effect have sometimes failed. The goal of this study is to investigate self-reported replication rates and the frequency of a set of questionable research practices (QRP) in ego depletion research. A literature search resulted in 1721 researchers who had previously published on ego depletion. They were invited to participate in an anonymous online survey. The respondents (*n* = 277), on average, had published over three papers on ego depletion, and had completed more than two additional, unpublished studies. Respondents indicated that in more than 40% of their studies, results were similar in magnitude to those reported in the existing literature, and more than 60% reported conducting a priori power analyses. 39.2% of respondents were aware of other researchers who engaged in the surveyed QRP’s, while 37.7% affirmed to have employed said QRP’s. These results underline the importance of reducing QRP’s to reliably test the validity of the ego depletion effect.

## Introduction

The strength model of self-control is one of the most cited research paradigms in social psychology [1].It defines self-control as a process that enables an individual to volitionally override behavioral impulses or dominant response tendencies (e.g., eating a cookie), and thus facilitates the achievement of long-term goals (e.g., healthy nutrition) [2]. According to Baumeister et al. [1], all self-control acts are based on one global resource. This self-control strength is assumed to have a limited capacity, meaning that it can become temporarily depleted after having exerted self-control. A temporary loss of self-control strength (ego depletion) negatively affects performance in subsequent self-control tasks[3].

The ego depletion effect is primarily investigated with the sequential two-task paradigm [1]: Participants are randomly assigned to an experimental condition or a control condition. The demands of the first task are similar in both conditions (e.g., watching an emotional video clip), but self-control strength is required only in the experimental condition (e.g., emotional suppression while watching the video clip) [4]. The subsequent secondary task is identical in both conditions and requires self-control (e.g., the Stroop task) [5]. It is assumed that participants in the control condition should outperform participants from the experimental condition in the secondary task, as their resources have not been taxed in the primary task. There have been plenty of studies supporting the ego depletion effect, and a meta-analysis by Hagger, Wood, Stiff, and Chatzisarantis (2010) found a medium-to-large effect size of ego depletion [6]. Additionally, a recently published meta-analysis by Dang [7] also found sound empirical evidence for the ego depletion effect.

Even though most ego depletion studies have supported the strength model’s propositions, there are some recent studies which have found no empirical evidence for the ego depletion effect [8,9]. Furthermore, a re-analysis of the findings by Hagger et al. [6] indicates that this effect size might reflect a case of publication bias which may have caused an overestimation of the true ego depletion effect [10–12].

These inconsistent findings and the general replication crisis in psychological science [13] led Hagger et al. [14] to initiate a Registered Replication Report (RRR), which included 23 labs and more than 2,000 participants. The authors tested the assumptions of the strength model with a standardized and fully computerized ego-depletion protocol [15]. Participants were randomly assigned to a control condition or a depletion condition and worked on the modified letter *e* task to manipulate self-control strength [3]. Then, all participants worked on a secondary task, the multisource interference task (MSIT) [16], which required self-control from all participants. According to the strength model [1], participants from the depletion condition should have performed significantly worse in this secondary task compared to participants from the control condition. However, the RRR failed to find a significant ego depletion effect. Hagger et al. [14] stress that the results do not imply that the ego depletion effect does not exist, but that it is highly necessary to identify the potential causes for these null-findings.

These unexpected results have instigated debate regarding the validity of the strength model [17]. For instance, Baumeister and Vohs [18] published a response letter in which they argue that the tasks applied in the RRR were not appropriate to test the strength model and that “in retrospect, the decision to use new, mostly untested procedures for a large replication project was foolish” (p. 574) [19]. It has also been argued that the general experimental setup chosen in the RRR was not appropriate to test the validity of the strength model [18]. It needs to be pointed out that the protocol was vetted by Baumeister [15] and the tasks applied in the RRR were adopted from a previous study which found empirical evidence for the ego depletion effect [15]. Still, it is conceivable that whether or not an ego depletion effect is found, seems to largely depend on the chosen tasks. Furthermore, the RRR data have been reanalyzed by applying alternative statistical methods or by focusing on certain subsamples (e.g., only English-speaking labs), which led to different but still non-significant results [20–22].

The recent discussions regarding the strength model have led us to shift our focus from testing the strength model’s validity to asking researchers that have contributed to ego depletion research about their experiences and research practices in the field of ego depletion. We would like to stress that we are not taking a stand regarding the validity of the strength models’ propositions. Our research’s focus is to provide a “behind the scenes” look into the current state of research on ego depletion as reported by ego depletion researchers. Such a behind the scenes look is highly necessary because—as recently stressed by Nelson, Simmons, and Simonsohn—“psychologists spent several decades relying on methods of data collection and analysis that make it too easy to publish false-positive, nonreplicable results” [23] (p 512). The inconsistencies regarding the ego depletion effect might also be a product of this scientific approach [24]. In recent years, psychological research has gone to great lengths to assess how frequently questionable research practices (QRP; e.g. selective reporting of subgroups or outlier rejection without statistical support), that might eventually lead to the publication of false-positive results, are employed [23]. A recent systematic review on QRP’s in the field of social sciences found substantial evidence for QRP’s in 91% of the analyzed studies [25]. Therefore, QRP’s appear to be a serious issue. However, some evidence suggests that methodological issues might cause an overestimation of QRP’s [26]. On the other hand, socially desirable responding might also affect QRP-reporting [27]. Addressing the use of QRP’s–which mostly does not represent an ill-intended act of data manipulation [23]–has led to ‘Psychology’s Renaissance’ and an improvement of research practices [23]. In light of the heated debate regarding the validity of the ego depletion effect, we believe it is paramount to directly ask relevant researchers on their research practices and experiences.

In order to accomplish this goal, we mirrored the approach by Héroux, Taylor, and Gandevia [28], who recently surveyed researchers to investigate practices and experiences regarding the effects of transcranial magnetic stimulation (TMS). Although TMS refers to a technique and ego depletion is a proposed psychological state, there are similarities: TMS and ego depletion have garnered substantial scientific and public attention, and, like the ego depletion effect, TMS effects have been difficult to replicate [28]. Héroux et al. found that 50% of their 47 respondents were able to reproduce TMS effects [28]. Furthermore, while only 18% affirmed applying QRP’s, 44% reportedly knew of researchers who applied such practices. In a more recent study, Héroux and colleagues reported similar results for electrical brain stimulation [29].

We conducted a literature search to identify researchers who had previously published on ego depletion. We invited all researchers of whom we were able to find a publicly available email-address to participate in an anonymized online survey that focused on their replication success of the ego depletion effect and QRP’s. More specifically, the survey focused on the number of published and unpublished studies on ego depletion per researcher, the rate at which researchers were able to find the ego depletion effect as a function of the ego depletion task employed in a given study, and the decisions made when no significant effects were found. Additionally, researchers were surveyed on their use and their knowledge of others’ use of QRP’s in the field of ego depletion. Following Héroux and colleagues, QRP’s are defined as “an unhealthy flexibility in collecting and analyzing research data” [28] (p. 6).

## Materials and methods

### Ethics statement

This study has been approved by the local ethics committee of the faculty of Human Sciences at the University of Bern, Switzerland. The study was carried out in accordance with the Declaration of Helsinki and the ethical guidelines for experimental research with human participants as proposed by the German Psychological Society (DGPs) and the American Psychological Association (APA). All persons gave their informed consent prior to their inclusion in the study.

### Procedure

To identify the researchers who had previously published on ego depletion in scientific journals, a literature search was conducted in PubMed and PMC in July 2017 (search terms “ego depletion”, “ego-depletion”, and “egodepletion”). We also extracted all the authors whose studies had been included in the meta-analysis by Hagger et al. [6] and all the authors who participated in the Registered Replication Report [14]. This led to a total of 2094 researchers and 1721 researchers whose email addresses could be identified (see also S1 File). It is important to note that this number is likely a substantial overestimation of the number of active ego depletion researchers. Email addresses were collected from all authors listed in each publication. It is therefore likely that many had left academia, changed fields, or contributed to the publications in ways that are not directly related to ego depletion (e.g., by conducting the statistical analyses or collecting neuroimaging data). An email link to an anonymous online survey was sent out to all researchers, inviting them to participate. The respondents who entered the online survey were informed about the purpose of the study, delivered informed consent and confirmed that they voluntarily agreed to participate.

On the first page of the online survey, respondents were asked to report their research area, the number of years they had been working in the field of ego depletion, the number of published and unpublished studies on ego depletion they had conducted, and how they usually determine their sample sizes (S2 File; for full protocol, see also https://www.protocols.io/view/s2-file-full-protocol-mf9c3r6).

Next, respondents were presented with seven of the most commonly used ego depletion tasks and asked which they had used in their own research. The tasks were the Stroop task [30], the attention control video [31], the letter *e* task [3], the transcription task [32], the N-back task [30], MSIT [16], and the emotional suppression video [33]. The respondents also had the opportunity to name any other tasks they had applied in their work on ego depletion. For each of the tasks they had previously used, respondents indicated whether they had been able to reproduce a similar effect for the respective task as reported in the original literature and what steps they took if they had not been able to reproduce the effect.

Next, questionable research practices were identified by asking respondents to provide information on how they generally conduct and report studies on ego depletion and how they think other ego depletion researchers conduct and report studies [28] (S2 File). Finally, respondents had the opportunity to provide any additional comments.

## Results

Out of the 1721 invited researchers, 119 could not be contacted due to mail delivery failures. A total of 379 researchers started the study, and a total of 277 worked through the survey to the end. Respondents were always given the option to skip any question, so the results below pertain to different subsamples, depending on whether or not respondents chose to skip a question. To increase the transparency of the results, we indicate for each question how many researchers answered (S3 file for full data set; https://figshare.com/articles/Survey_sav/5759520).

The 277 respondents worked in the following research areas: *n* = 242 in psychology (87.4%), *n* = 40 in neuroscience (14.4%), *n* = 15 in sport science (5.1%), *n* = 3 in clinical neurology (1.1%), *n* = 5 in neurophysiology (1.8%), *n* = 40 in other sciences (14.4%). Within the “other” domain, organizational behavior was the most prominent occupation (*n* = 5, 1.8%; S4 File). Respondents (*n* = 190) had been working in the field of ego depletion for on average 5.43 years (*SD* = 4.68) and had published on average 3.4 (*n* = 183) papers in scientific journals (*SD* = 7.48). Furthermore, respondents (*n* = 182) on average had completed 2.68 additional studies that they did not publish (*SD* = 4.13). The number of published and unpublished studies and papers per researcher is highly correlated, *r* = .63, *p* < .001, CI95% = .53 - .71. Thus, the more studies a researcher had published on ego depletion, the more studies on the topic he or she had not yet published.

Most of the *n* = 176 respondents had previously applied more than one ego depletion task in their research. From the frequently used ego depletion tasks, 85 had used the letter *e* task (48.3%), 62 had used the Stroop task (35.2%), 41 had used the emotion suppression task (23.3%), 38 had used attention control video (21.6%), 18 had used N-back (10.2%), 16 had used the transcription task (9.1%), and 15 had used the MSIT (8.5%). The provided categories were not exhaustive as 70 (39.8%) reported to have used other ego depletion tasks.

Respondents (*n* = 190) had used several different methods in the past to determine sample sizes. 121 (63.7%) of them reported that they had conducted a priori power analyses based on previously published studies and 77 (40.5%) had determined their sample sizes based on their personal experiences. Some respondents deviated from their predetermined sample sizes. 13 (6.8%) had collected additional subjects if needed or stopped data collection either because a clear effect (*n* = 5; 2.6%) or no clear effect (*n* = 7; 3.7%) was noted. Finally, 5 respondents (2.6%) reported to have determined their sample sizes based on the appearance of the data.

Out of *n* = 253 respondents, 107 (42.29%) reported to have found (on average) similar results to those reported in the original literature when using the respective ego depletion tasks in their research. The replication rate varied between 33.3% for the letter *e* task) and 54.1% for the emotion suppression task. Even if the published effect was not found, many researchers still tried to publish findings while reporting the inability to reproduce the published effect. The full results for these questions are displayed in Table 1.

**Table 1:** Primary self-control tasks: Were you able to reproduce an effect similar to the one reported in the literature?

	Some-times		If „Yes“–how big was the effect compared to the literature?			If “No”–what steps did you take? Please select all that apply (Q1-Q6)						
			Ʃ	<	=	>	Ʃ	Q1	Q2	Q3	Q4	Q5	Q6
	n (%)	n (%)	n (%)	n (%)		n (%)	n (%)	n (%)	n (%)		n (%)	n (%)	n (%)	n (%)
Letter e	18 (22,2%)	**27 (33,3%)**	6 (26,1%)	16 (69,6%)	1 (4,3%)	**36 (44,4%)**	3 (8,6%)	2 (5,7%)	2 (5,7%)	14 (40,0%)	7 (20,0%)	16 (45,7%)
Stroop	13 (21,3%)	**25 (41,0%)**	4 (17,4%)	17 (73,9%)	2 (8,7%)	**23 (37,7%)**	5 (22,7%)	0	1 (4,5%)	7 (31,8%)	10 (45,5%)	7 (31,8%)
Emotion Suppres-sion	7 (18,9%)	**20 (54,1%)**	3 (15,0%)	17 (85,0%)	0	**10 (27,0%)**	1 (10,0%)	0	0	2 (20,0%)	3 (30,0%)	5 (50,0%)
Attention Control	9 (25,0%)	**15 (41,7%)**	5 (35,7%)	9 (64,3%)	0	**12 (33,3%)**	1 (8,3%)	0	0	3 (25,0%)	2 (16,7%)	9 (75,0%)
n-back Task	2 (11,8%)	**7 (41,2%)**	3 (42,9%)	4 (57,1%)	0	**8 (47,1%)**	1 (12,5%)	0	0	4 (50,0%)	3 (37,5%)	2 (25,0%)
Transcription Task	1 (6,7%)	**8** **(53,3%)**	3 (37,0%)	5 (62,5%)	0	**6 (40,0%)**	0	0	0	4 (66,7%)	0	1 (16,7%)
MSIT^a^	2 (15,4%)	**5 (38,5%)**	n/a^b^	n/a^b^^b^	n/a^b^	**6 (46,2%)**	1 (16,7%)	0	0	0	0	6 (100%)

*Note*. Q1 = Collect data from a greater number of subjects; Q2 = Select a subset of subjects that were "susceptible" to the investigated effect; Q3 = Contact the original authors for clarification; Q4 = Stop the assessment; Q5 = Modify the ego depletion task; Q6 = Try to publish the finding stating that you were unable to reproduce the published effect.

^*a*^ *=* Multi Source Interference Task

^b^ = no analyzable data due to survey malfunction.

**Fig 1:**
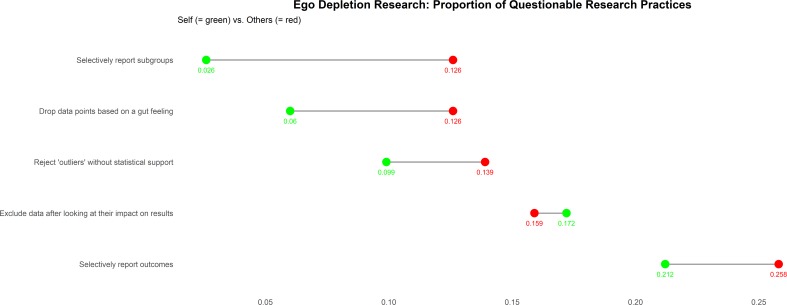
Dumbbell plot. Dumbbell plot visualizing the proportion of questionable research practices.

Fig 1 displays the data on QRP’s as conceptualized by Héroux et al. [28]. The percentages of respondents who affirmed to have used QRP’s varied between 2.6% and 21.2%, depending on the research practice in question. These values are slightly higher, when respondents were asked if they were aware of other researchers who had used such practices, 12.6% - 25.8%. The most frequently applied questionable research practice was to selectively report outcomes. Still, an overwhelming majority of 94.3% of respondents stressed that questionable research practices should be reported when publishing.

## Discussion

Based on the current debate regarding the ego depletion effect [8–10,12,14] and the heated discussion surrounding the strength model [18], this study aimed at collecting information on replication rates and QRP’s in the field of ego depletion. To gather this information, researchers who had previously published on ego depletion were invited to participate in an anonymous online survey. Researchers in our dataset had published, on average, three studies on ego depletion and had completed an additional two unpublished studies on ego depletion. The number of published and unpublished studies was highly correlated, suggesting replication rates that are similar for different researchers. This speaks against the idea that some researchers are *always* successful (or unsuccessful) in their ego depletion studies. In fact, the proportion of published and unpublished studies aligns with the findings by Carter and McCullough who suggested that the ego depletion effect is overestimated and that this overestimation is likely caused by publication bias [24]. However, it is important to note, that it cannot be concluded that none of the unpublished studies found evidence for the ego depletion effect. It might also be possible that these studies did not get published for other reasons. However, Carter and McCullough’s analyses reveal a striking lack of non-significant findings in published ego depletion research [24], which hints at a body of unpublished non-significant findings. Indeed, our findings suggest, that a large body of grey literature on ego depletion exists [34] and further research is needed to analyze these currently unpublished studies.

The respondents indicated that in over 40% of their studies the results were similar in magnitude to those reported in the original literature when using the respective ego depletion tasks. Interestingly, this replication rate varied substantially between the tasks used. While only 27% of researchers who had used the emotion suppression task did not find the expected effect, 47.1% who had used the n-back task failed to replicate the initial effect. Thus, whether or not an ego depletion effect is found also depends on the chosen self-control task and even well-established tasks differ substantially in terms of their replication rate. More than 60% of respondents reported that they had applied a priori power analyses to determine the required sample sizes, which is a high value compared to other fields. For instance, in the aforementioned study by Héroux et al. [28], only 20% of respondents conducted a priori power analyses.

In this study, 39.2% of respondents were aware of other ego depletion researchers who had applied QRP’s and 37.7% affirmed that they had previously applied QRP’s. Compared to the findings by Héroux et al. [28], who investigated questionable research practices regarding the application of TMS, our numbers are substantially lower. Compared to other studies on QRP’s in psychology, albeit assessing larger number of different QRP’s, our numbers are descriptively lower too [35]. In addition, although respondents were aware of more QRP’s among others than among themselves, this difference is comparatively small. Taken together, our self-report data do not reveal an unusually high engagement with the assessed QRP’s among ego depletion researchers. If anything, the QRP frequencies and the discrepancy between the self-other evaluations regarding QRP engagement we observed are comparatively minor [28].

## Limitations

Finally, some limitations of this study should be addressed. First, even though the response rate was high in comparison to similar online surveys [28,36], selection bias cannot be ruled out [37]. For instance, it might be possible that only researchers who question the propositions of the strength model completed the survey. On the other hand, it is also reasonable that those researchers who took part in the survey were those who truly believe in the ego depletion effect and want to defend the strength model. In addition, engaging in QRP’s is a socially sensitive topic [38] and it is possible that researchers who have engaged in QRP’s were less likely to take part in the survey. Taken together, selection bias cannot be ruled out, which is why we cannot infer that our findings represent the opinions and research approaches of the entire ego depletion community. However, this applies to all research that relies on self-selected samples. We believe that in order to advance our understanding of self-control, it is crucial to survey and report the personal experiences and practices of those who are willing to contribute.

Second, although it has been used in previous research [28] the questionnaire used in this study is not without limitations. Most importantly, we did not survey an exhaustive list of possible QRP’s, but only a limited subset [28]. Still, there are multiple reasons why we chose to emulate the approach by Héroux et al. [28]. Both researches aimed at surveying a small and specified population (i.e., ‘ego depletion’ or ‘TMS’ researchers as opposed to ‘all members of the German society for psychology’ [26], which is why it was paramount to lose as few respondents as possible. As longer questionnaires tend to yield lower response rates [39], we decided to assess a lower number of QRPs in order to increase the number of respondents. Furthermore, research on QRP’s has burgeoned in recent years but a gold standard QRP-questionnaire has yet to be established. In order to allow for comparisons between studies, we decided to use a questionnaire that had already been used in two highly published studies. Third, the fields surveyed by Héroux et al.–like ego depletion research–are very popular but are also controversially debated. This might make it even harder to convince researchers to participate, compared to surveys that are distributed among a broader population of researchers. As Héroux et al. were able to achieve sufficient sample sizes with their approach and provide valuable findings, we decided to mirror it as it has proven successful under these conditions. Still, these limitations should not be ignored and going forward, an important next step is to standardize research on QRP’s in order to increase the usefulness of such findings and to facilitate comparisons between studies.

Third, several researchers responded via email with reasons why they did not want to participate in the survey. For instance, some researchers had the impression that the study was merely a witch-hunt, while others stated that is more important to develop and adjust the strength model than conduct further studies on its validity. However, it is important to repeat that this study does not take a position in favor or against the strength model. The aim is to present common research practices and experiences in the field of ego depletion research, without giving any recommendations or drawing any conclusion regarding the effects’ validity. Up until now, such a survey among ego depletion researchers has never been conducted, as previous studies have been more interested in testing the ego depletion effect at an empirical level [14]. That is why this study provides a novel, valuable insight into the current state of ego depletion research.

## Conclusion

The results of this survey suggest, that it seems highly necessary to critically reflect on the current research practices. For example, when applying the sequential two-task paradigm [3] there needs to be a common standard for how long the primary self-control task should last. In some studies, participants had to work on fewer than 50 Stroop trials [40], while in other studies participants were asked to perform more than 250 trials [41]. Considering these drastic methodological differences, the inconsistent findings on ego depletion are not surprising. It should also be noted that it is not clear how long it takes until supposedly depleted self-control strength is fully recovered. This is a major problem as it determines the necessary timeframe between completing a primary task and starting the secondary task. In some studies, there is a break of several minutes between the two tasks [42], while in others there is no break at all [3]. Again, to reliably compare different ego depletion studies, there needs to be a general agreement regarding the research approach [43].

Furthermore, most studies analyze self-control performance by comparing the mean values of the depletion and the control condition (e.g., number of correctly answered Stroop trials) [30]. However, recent studies using multilevel modeling indicate that ego depletion might indeed lead to progressive performance decreases over time. This could indicate that the ego depletion effect is stronger towards the end of a self-control task [44]. Based on these findings, future studies should consider to analyze ego depletion effects over time instead of simply comparing mean values.

## Supporting information

S1 File Flow chart of the search strategy. (PNG)

S2 File Study protocol. https://www.protocols.io/view/s2-file-full-protocol-mf9c3r6.
(PDF)

S3 File Full data set. https://figshare.com/articles/Survey_sav/5759520.
(SAV)

S4 File Occupations. (PDF)
