# Supplementary figures and images for: Self-reports from behind the scenes: Questionable research practices and rates of replication in ego depletion research
Source: PLoS One. 2018 Jun 25;13(6):e0199554. doi: 10.1371/journal.pone.0199554 (PMC6016937; doi:10.1371/journal.pone.0199554)

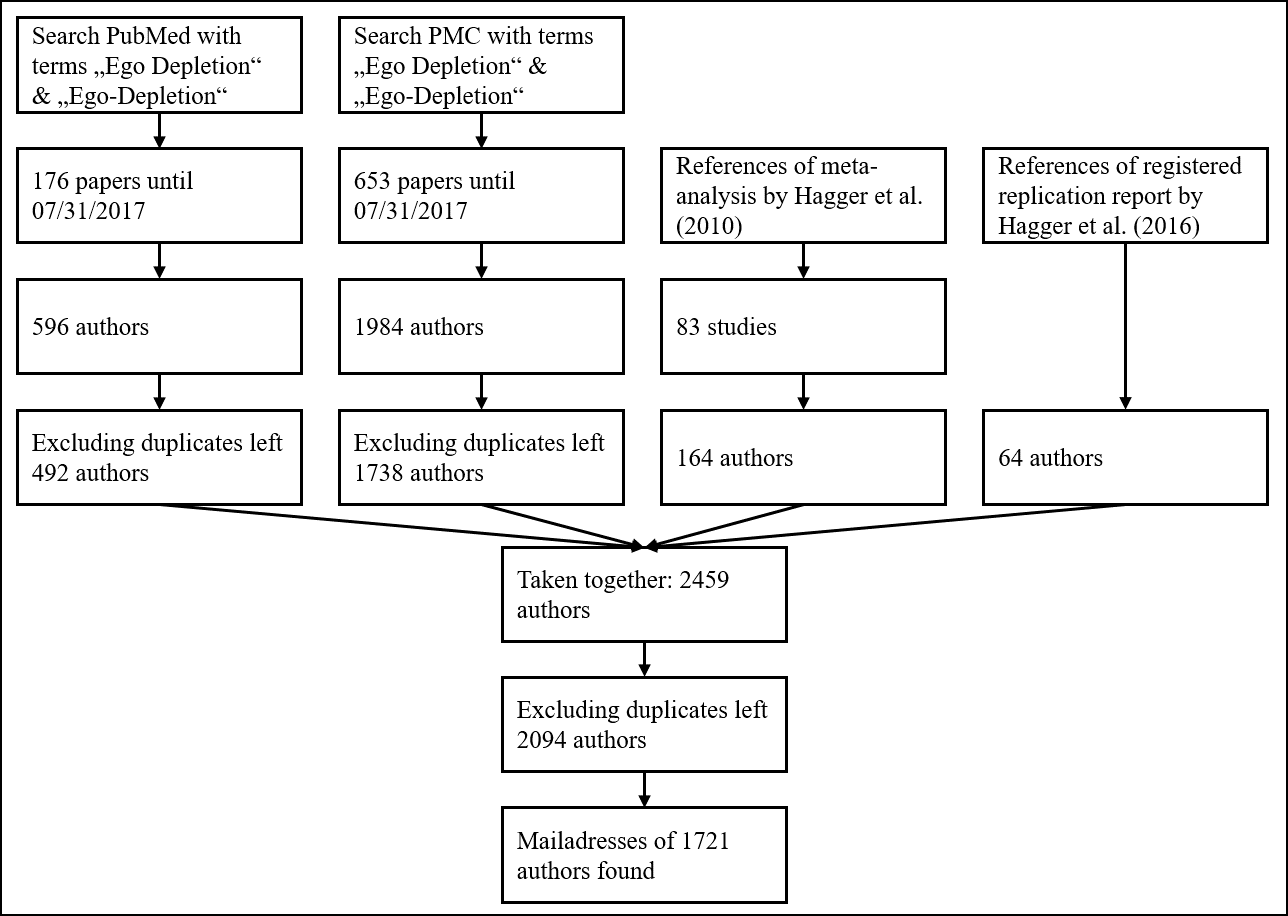

Supplement: S1 File — (PNG) [file pone.0199554.s001.png]
