# Supplementary material for: Self-reports from behind the scenes: Questionable research practices and rates of replication in ego depletion research
Source: PLoS One. 2018 Jun 25;13(6):e0199554. doi: 10.1371/journal.pone.0199554 (PMC6016937; doi:10.1371/journal.pone.0199554)
Supplement: S2 File — https://www.protocols.io/view/s2-file-full-protocol-mf9c3r6. (PDF) [file pone.0199554.s002.pdf]

Article title: The Strength Model of Self-Control: Research Practice and Experience

Author names: Chris Englert, Wanja Wolff & Lorena Baumann

Corresponding author: Chris Englert, University of Bern, Institute of Educational Science, Department of Educational Psychology, Fabrikstrasse 8, 3012 Bern, Switzerland, Phone: +41 (0)31 631 8275, Email: christoph.englert@edu.unibe.ch

Dear colleagues,

thank you for your participation in this survey. The following questions are concerned with your experience in working on the ego depletion effect.

Your participation in this study is voluntary. However, given the recent controversy surrounding the ego depletion effect, we would appreciate your support! You may choose to not answer any of the questions or withdraw from this study at any time. All information collected will **remain anonymous** and will only be subjected to group level analysis. Individual responses will not be identifiable at any time.

By entering this survey, you are indicating that you have read this consent form, you are age 18 or older and that you voluntarily agree to participate in this study.

What area of research do you work in?
Please select all that apply.

☐	Psychology
☐	Neuroscience
☐	Sport Science
☐	Clinical neurology
☐	Neurophysiology
☐	Other ______

Approximately how long have you been working in the field of ego depletion?
(Number of Years)

[drop-down menu with 30 options, ranging from 1 year to 30 years]

Approximately how many studies on ego depletion have you published in peer-reviewed journals?

______ Studies

How many of these studies have specifically involved an experimental manipulation of self-control strength by applying the two-task paradigm?

______ Studies

Approximately how many studies on ego depletion have you completed but not yet published?

______ Studies

How did you determine the sample size of your study/studies on ego depletion?
Please select all sampling strategies that you have used.

☐	Using previously published results to perform a power calculation and estimating sample size requirements
☐	Based on prior personal experience
☐	Based on the appearance of the data
☐	Prior to starting the study, but stopping early if no clear effect is noted
☐	Prior to starting the study, but allowing for additional subjects to be included if needed
☐	Prior to starting the study, but stopping early if a clear effect is noted

---

Have you used any of the following as a primary self-control task to experimentally manipulate ego depletion?

☐	Stroop task (e.g., Webb & Sheeran, 2003)
☐	Attention control video (e.g., Schmeichel et al., 2003)
☐	Letter "e" task (e.g., Baumeister et al., 1998)
☐	Transcription task (e.g., Bertrams et al., 2010)
☐	N-back task (e.g., Webb & Sheeran, 2003)
☐	Emotion suppression while watching a video clip (e.g., Muraven et al., 2008)
☐	Multi-source interference task (MSIT; e.g., Bush et al., 2003)
☐	Other, please specify: ________

When using the Stroop task (e.g., Webb & Sheeran, 2003), have you (on average) been able to reproduce a similar effect to the one reported in the original literature?

○	Yes
○	No
○	Sometimes

When using the Attention Control Video (e.g., Schmeichel et al., 2003), have you (on average) been able to reproduce a similar effect to the one reported in the original literature?

○	Yes
○	No
○	Sometimes

When using the Transcription Task (e.g., Bertrams et al., 2010), have you (on average) been able to reproduce a similar effect to the one reported in the original literature?

○	Yes
○	No
○	Sometimes

When using the N-back Task (e.g., Webb & Sheeran, 2003), have you (on average) been able to reproduce a similar effect to the one reported in the original literature?

○	Yes
○	No
○	Sometimes

When using Emotion suppression while watching a video clip (e.g., Muraven et al., 2008), have you (on average) been able to reproduce a similar effect to the one reported in the original literature?

○	Yes
○	No
○	Sometimes

When using the Multi-Source Interference Task (MSIT; e.g., Bush et al., 2003), have you (on average) been able to reproduce a similar effect to the one reported in the original literature?

○	Yes
○	No
○	Sometimes

When using the additionally specified primary self-control task, have you (on average) been able to reproduce a similar effect to the one reported in the original literature?

○	Yes
○	No
○	Sometimes

If "No" has been selected:
If you were not able to reproduce an effect, what steps did you take? Please select all and apply.

☐	Collect data from a greater number of subjects

☐	Select a subset of subjects that were "susceptible" to the investigated effect
☐	Contact the original authors for clarification
☐	Stop the assessment
☐	Modify the ego depletion task
☐	Try to publish the finding stating that you were unable to reproduce the published effect

---

If “Yes” has been selected:
If you were able to reproduce an effect, was it (on average) similar in magnitude to the originally published effects?

○	Yes
○	No, the effect found in our laboratory was smaller
○	No, the effect found in our laboratory was larger

If “Sometimes” has been selected:
Please explain:

Please answer "Yes" or "No" in regard to research on ego depletion. Please remember that your responses will remain anonymous and will only be subjected to group level analysis. Individual responses will not be identifiable at any time! We appreciate your support to improve our knowledge regarding the ego depletion effect.
Are you aware of other ego depletion researchers who have discontinued observations or data points from their analyses based on a gut feeling that they were inaccurate?

○	Yes
○	No

Are you aware of other researchers who have decided to exclude data after examining the impact of doing so on the results?

○	Yes
○	No

Are you aware of other researchers who decided post-hoc to selectively report outcomes in research publications? (without acknowledging this post-hoc decision in their paper)

◯	Yes
◯	No

Are you aware of other researchers who decided post-hoc to selectively report sub-groups of subjects in research publications? (without acknowledging this post-hoc decision in their paper)

◯	Yes
◯	No

Are you aware of researchers who visually inspect their data and reject trials or subjects deemed to be "outliers" without the support of statistical analysis?

◯	Yes
◯	No

Should these various practices be reported by researchers when they publish their research results?

◯	Yes
◯	No

Please answer "Yes" or "No" in regard to research on ego depletion. Please remember that your responses will remain anonymous and will only be subjected to group level analysis. Individual responses will not be identifiable at any time! We appreciate your support to improve our knowledge regarding the ego depletion effect.

Have you ever discontinued observations or data points from analyses based on a gut feeling that they were inaccurate?

◯	Yes
◯	No

Have you ever decided to exclude data after looking at the impact of doing so on the results?

◯	Yes
◯	No

Have you ever decided post-hoc to not report all experimental conditions of a study in research publications? (without acknowledging this post-hoc decision in your paper)

◯	Yes
◯	No

Have you ever decided post-hoc to selectively report outcomes in research publications? (without acknowledging this post-hoc decision in your paper)

◯	Yes
◯	No

Have you ever decided post-hoc to selectively report sub-groups of subjects in research publications? (without acknowledging this post-hoc decision in their paper)

○	Yes
○	No

Have you ever visually inspected data and rejected trials or subjects deemed to be "outliers" without the support of statistical analyses?

○	Yes
○	No

Thank you for your participation in this survey. If you have any additional comments, please use the box provided below:
