# Supplementary material for: Self-reports from behind the scenes: Questionable research practices and rates of replication in ego depletion research
Source: PLoS One. 2018 Jun 25;13(6):e0199554. doi: 10.1371/journal.pone.0199554 (PMC6016937; doi:10.1371/journal.pone.0199554)
Supplement: S4 File — (PDF) [file pone.0199554.s004.pdf]

Article title: The Strength Model of Self-Control: Research Practice and Experience

Author names: Chris Englert, Wanja Wolff & Lorena Baumann

Corresponding author: Chris Englert, University of Bern, Institute of Educational Science, Department of Educational Psychology, Fabrikstrasse 8, 3012 Bern, Switzerland, Phone: +41 (0)31 631 8275, Email: christoph.englert@edu.unibe.ch

Field of profession	Number of respondents
Academic Administration/Graduate Education	1
Applied Statistics	1
Assessment of Organizations	1
Behavioural Economics	2
Behavioural Medicine	1
Communication	1
Computer Science	1
Education/Educational Research	2
Health Behaviour/Sciences	2
Information Science	1
Management	2
Marketing	4
Motivation Science	1
Neuroethics	1
Organizational Behaviour	6
Philosophy	1
Public Health	5
Quantitative Psychology	1
